# Time-course analysis of *Streptococcus sanguinis* after manganese depletion reveals changes in glycolytic and nucleic acid metabolites

**DOI:** 10.1007/s11306-021-01795-2

**Published:** 2021-04-23

**Authors:** Tanya Puccio, Biswapriya B. Misra, Todd Kitten

**Affiliations:** 1 Philips Institute for Oral Health Research, Virginia Commonwealth University School of Dentistry, Richmond, VA 23298 USA; 2 Department of Internal Medicine, Section on Molecular Medicine, Wake Forest School of Medicine, Medical Center Boulevard, Winston-Salem, NC 27157 USA

**Keywords:** Metabolomics, Manganese, Endocarditis, Multivariate, Time-course

## Abstract

**Introduction:**

Manganese is important for the endocarditis pathogen *Streptococcus sanguinis*. Little is known about why manganese is required for virulence or how it impacts the metabolome of streptococci.

**Objectives:**

We applied untargeted metabolomics to cells and media to understand temporal changes resulting from manganese depletion.

**Methods:**

EDTA was added to a *S. sanguinis* manganese-transporter mutant in aerobic fermentor conditions. Cell and media samples were collected pre- and post-EDTA treatment. Metabolomics data were generated using positive and negative modes of data acquisition on an LC–MS/MS system. Data were subjected to statistical processing using MetaboAnalyst and time-course analysis using Short Time series Expression Miner (STEM). Recombinant enzymes were assayed for metal dependence.

**Results:**

We observed quantitative changes in 534 and 422 metabolites in cells and media, respectively, after EDTA addition. The 173 cellular metabolites identified as significantly different indicated enrichment of purine and pyrimidine metabolism. Further multivariate analysis revealed that the top 15 cellular metabolites belonged primarily to lipids and redox metabolites. The STEM analysis revealed global changes in cells and media in comparable metabolic pathways. Glycolytic intermediates such as fructose-1,6-bisphosphate increased, suggesting that enzymes that utilize them require manganese for activity or expression. Recombinant enzymes were confirmed to utilize manganese in vitro. Nucleosides accumulated, possibly due to a blockage in conversion to nucleobases resulting from manganese-dependent regulation.

**Conclusion:**

Differential analysis of metabolites revealed the activation of a number of metabolic pathways in response to manganese depletion, many of which are connected to carbon catabolite repression.

**Supplementary Information:**

The online version contains supplementary material available at 10.1007/s11306-021-01795-2.

## Introduction

*Streptococcus sanguinis* is a gram-positive bacterium known for its duplicity. As an early and abundant colonizer of teeth, *S. sanguinis* is associated with oral health (Kreth et al., 2005, 2017). However, when it enters the bloodstream, whether through dental procedures or activities as routine as eating, it is known to colonize the heart valves or other endocardial surfaces of persons with particular pre-existing cardiac conditions, leading to infective endocarditis (IE) (Moreillon et al., 2002; Widmer et al., 2006). IE has a global mortality rate of 12–40% (Bor et al., 2013; Cahill et al., 2017). Historically, prevention has relied upon administration of prophylactic broad-spectrum antibiotics to high-risk patients prior to dental visits (Wilson et al., 2007). With rising antibiotic resistance (Dodds, 2017), as well as controversial efficacy (Quan et al., 2020; Thornhill et al., 2018), novel drug targets that are required for endocarditis causation but not beneficial colonization are under investigation.

One such potential drug target in *S. sanguinis* is the lipoprotein SsaB, a component of the ATP-binding cassette transporter SsaACB. This transporter and orthologs in related species have been shown to be important for manganese (Mn) transport and virulence (Colomer-Winter et al., 2018; Crump et al., 2014; Dintilhac et al., 1997; Kehl-Fie et al., 2013). Previous studies utilizing a Δ*ssaACB* strain of *S. sanguinis* revealed that this mutant is deficient in cellular Mn (Murgas et al., 2020) and virulence in our rabbit model of IE (Baker et al., 2019). These studies also suggested that the reduced virulence of Mn-deficient cells is due to growth arrest in the aerobic, low-Mn environment characteristic of an aortic valve infection, implying the existence of one or more Mn-dependent metabolic pathways that are essential for aerobic growth. The metabolic pathway(s) and individual metabolites involved have not been defined.

Metabolomics is the comprehensive study of small molecules in the molecular weight range of 50–2000 Da in biological systems. Diverse mass spectrometry platforms such as LC–MS/MS, GC–MS and CE–MS with and without chromatography, and spectroscopy technologies such as NMR have enabled high-throughput discovery metabolomics in various biological systems, including bacteria, plants, and humans (Misra & Olivier, 2020). Recent studies have described the metabolomes of certain streptococci using various mass spectrometry methods: *Streptococcus intermedius* under various oxygen conditions (Fei et al., 2016); *Streptococcus pneumoniae* in chemically defined medium (Leonard et al., 2018); and *Streptococcus thermophilus* in pH-controlled batch fermentation (Liu et al., 2020; Qiao et al., 2019). To our knowledge, the metabolome of *S. sanguinis* has yet to be investigated. Here we report the first untargeted metabolomic analysis of *S. sanguinis* or, indeed, of any *Streptococcus*, under Mn replete vs. deplete conditions.

## Materials and methods

### Bacterial strains and growth conditions

*S. sanguinis* strain SK36 was isolated from human dental plaque (Kilian et al., 1989; Xu et al., 2007). The Δ*ssaACB* strain (JFP169) was generated from SK36 previously by replacement of the *ssaACB* genes with the *aphA-3* gene encoding kanamycin resistance (Baker et al., 2019; Puccio et al., 2020). Overnight pre-cultures were created by inoculation of Brain Heart Infusion (BHI) broth (BD) with single-use aliquots of cryopreserved cells by 1000-fold dilution. Kanamycin (Sigma-Aldrich) was added to 500 µg mL^−1^ for Δ*ssaACB* pre-cultures. Pre-cultures were incubated at 37 °C for 18 h in 6% O_2_ (6% O_2_, 7% H_2_, 7% CO_2_ and 80% N_2_) using an Anoxomat (Advanced Instruments) jar.

### Fermentor growth conditions and sample collection

Aerobic fermentor growth of Δ*ssaACB* cell culture was achieved using a BIOSTAT® B bioreactor (Sartorius Stedim) and samples were collected as described in Puccio and Kitten (2020) and Puccio et al. (2020). Briefly, a 40-mL overnight pre-culture of *S. sanguinis* was grown as described above and centrifuged for 10 min at 3740×*g* at 4 °C (Beckman-Coulter). The supernatant was discarded and the cells were resuspended in BHI prior to inoculation into the 800 mL of BHI in the fermentor vessel. Cultures were stirred at 250 rpm at 37 °C and pH was maintained at 7.4 by the automated addition of 2 N KOH (Fisher Chemical). The air flow was increased stepwise, based on the OD of the fermentor culture. At the peak OD, air flow was increased to 0.50 lpm, input flow of BHI was set to 17% (~ 700 mL h^−1^) and output flow of waste was set to 34%. Cells were allowed to acclimate to the new conditions for 1 h. The T_-20_ sample was aseptically removed for metabolomics or ICP-OES analysis (described in Supplementary Methods). Approximately 2 × 10^10^ colony-forming units (CFU) were collected per 40 mL sample. EDTA (Invitrogen) was introduced to the media carboy 16 min later (T_-4_) to achieve a final concentration of 100 µM. EDTA was then introduced directly to the vessel 4 min later (T_0_), corresponding to the time at which EDTA from the carboy would reach the vessel, to achieve a final concentration of 100 µM. Samples were taken for each post-EDTA time point (T_25_, T_50_). All metabolomics samples were stored at − 80 °C until shipped on dry ice to Metabolon, Inc. (Durham, North Carolina) for further analysis. Supernatants were also collected from each time point and hydrogen peroxide (H_2_O_2_) levels were measured as described in the Supplementary Methods.

### Sample preparation, UPLC-MS/MS, data extraction, compound identification, and curation

Metabolomics sample processing was completed by Metabolon, Inc. as described in the Supplementary Methods and in previous publications (Dehaven et al., 2010; Evans et al., 2009).

### Activity of recombinant glycolytic enzymes

Recombinant proteins were expressed and purified as described in the Supplementary Methods. Aldolase activity was measured using a coupled spectrophotometric assay as described in Labbe et al. (2011). The assay mixture contained 0.3 mM NADH, 0.2 U mL^−1^ of rabbit muscle α-glycerophosphate dehydrogenase, 2.25 U mL^−1^ of rabbit muscle triose phosphate isomerase, 0.2 mg mL^−1^ BSA, 100 mM potassium acetate, and 50 mM Tris–HCl, pH 8.0. Recombinant *S. sanguinis* Fba (rFba) was incubated in 1 mM EDTA for 15 min at RT and added to the assay mixture at 5 µg mL^−1^ (10 µM EDTA final). Metals (Puratronic™; > 99.999% purity; Alfa Aesar) were added to respective wells at a final concentration of 1 mM. Assays were performed in 96-well flat bottom polystyrene plates (Grenier) with 100 µL volume. The plates were pre-warmed at 30 °C for 10 min prior to the addition of 1 mM fructose 1,6-bisphosphate (FBP). Wells were monitored at 340 nm for 10 min at 30 °C on a Synergy H1 plate reader (BioTek). One unit of aldolase activity is defined as the amount of enzyme required to cleave 1 pmol FBP per min at 30 °C; activity was detected by monitoring accumulation of NADH with an extinction coefficient of 6220.

Phosphatase activity was measured using a coupled spectrophotometric assay as described in Gutka et al. (2011). The assay mixture contained 50 mM potassium chloride, 20 mM tricine pH 7.70, 1.0 mM NADP+, yeast glucose-6-phosphate dehydrogenase (G6PDH; 1.0 U mL^−1^), and yeast phosphoglucoisomerase (PGI; 2.5 U mL^−1^). Recombinant *S. sanguinis* Fbp (rFbp) was incubated in 1 mM EDTA for 15 min at RT and added to the assay mixture at 12 µg mL^−1^ (10 µM EDTA). Metals (Puratronic™; > 99.999% purity; Alfa Aesar) were added to respective wells at a final concentration of 8 mM. Assays were pre-incubated, initiated, and measured as described for the aldolase assay. One unit of phosphatase activity is defined as the amount of enzyme that hydrolyzes 1 pmol FBP per min at 30 °C, detected as loss of NADPH using an extinction coefficient of 6220.

### Statistical analysis

Statistical analysis of the metabolomics data sets was performed using statistical software R (Version 3.5.2) (Team, 2018). Median normalized, cube root-transformed, KNN-imputed, outlier-removed, and scaled-peak (mean-centering) areas representative of relative metabolite amounts obtained using DeviumWeb (Grapov, 2014) are presented. Hierarchical clustering analysis (HCA) was performed on Pearson distances using MetaboAnalyst 4.0 (www.metaboanalyst.ca) (Xia et al., 2015), with the data normalized using z-scores of the relative abundance of the metabolites for heat map display. Correlations reported are Spearman rank correlations. Principal component analysis (PCA) and partial least squared discriminant analyses (PLS-DA) were performed using MetaboAnalyst, with the output displayed as score plots for visualization of sample groups. One-way analysis of variance (ANOVA) followed by post-hoc analysis using Fisher’s least significant difference (LSD) test was used for analysis of statistical significance using MetaboAnalyst.

Statistical analysis of the H_2_O_2_ measurements, metal analysis, and enzymatic assays were determined using a t-test or ANOVA with a Tukey multiple comparisons post-test in GraphPad Prism (version 9.0) as described in the figure legend.

## Results and discussion

### EDTA treatment of Δ*ssaACB* cells leads to Mn depletion and slowed growth

**Fig. 1:**
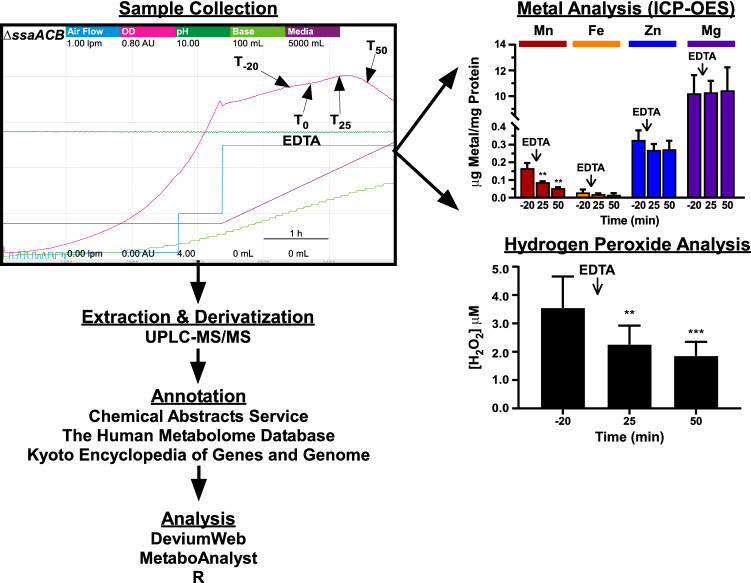
Overview of the experimental design, platform and software tools used for the analysis of metabolomic changes in cells and media subjected to EDTA treatment. For the sample collection chart, each color represents a different parameter and the minimum and maximum values are indicated by the values under each respective label. The time scale is indicated by the bar in the bottom right portion of the chart. Cells were grown under aerobic conditions with EDTA added 80 min (T_0_) after the media pumps were turned on and the air flow was set to 0.5 lpm. Each sample time point is labeled. Extraction, derivatization, and annotation were completed by Metabolon, Inc. ICP-OES, inductively coupled plasma optical emission spectroscopy; UPLC-MS/MS, ultra performance liquid chromatography with tandem mass spectrometry. Fermentor sample collection, metal analysis, and H_2_O_2_ analysis charts adapted from Puccio et al. (2020)

Our goal was to understand the metabolic consequences of Mn depletion during the growth of *S. sanguinis* in a rich medium (BHI), as well as to survey changes in the conditioned media during the growth and treatment periods. As we are unaware of the existence of a strong, specific chelator of Mn, we elected to use a Mn-transporter mutant of *S. sanguinis* in combination with the nonspecific metal chelator, EDTA. As described in Puccio et al. (2020), EDTA treatment of Δ*ssaACB* aerobic fermentor-grown cells results in the depletion of Mn but no other biologically relevant metals, such as Fe, Zn, or Mg, as determined by inductively coupled plasma optical emission spectroscopy (ICP-OES) (Fig. 1). Beginning ~ 38 min post-EDTA addition, cell growth slowed, resulting in a steady drop in OD (Fig. 1). We observed an immediate increase in OD after shutting off the media pumps (data not shown), despite the continued presence of EDTA in the media, indicating that the cells had not died but were unable to replicate fast enough to avoid dilution. Additionally, the cells were still viable by plating (Fig. S1 in Electronic Supplementary Material).

### Global metabolomics of ***S. sanguinis*** cells and BHI media

Extensive global untargeted metabolomics analysis revealed 534 metabolites in cells and 422 metabolites in conditioned media. The raw metabolite abundance values alongside the identified metabolite IDs, super pathways and sub-pathway names, average mass, and identifiers such as Chemical Abstracts Service (CAS), PubChem, Kyoto Encyclopedia of Genes and Genomes (KEGG), and Human Metabolome Database (HMDB) IDs are provided for both cellular and media metabolites (Tables S1, S2). These datasets were refined through normalization, transformation, and scaling, followed by imputation (Tables S3-S4). The 534 metabolites belong to 37 different BioCyc metabolic pathways (Table S5). The 422 metabolites quantified in the conditioned BHI media belonged to 40 different metabolic pathways (Table S6).

BHI has as its chief constituents bovine and porcine brain and heart extracts. We identified several metabolites that were found in all pre-inoculation media samples but in few of the later media or cell samples. These media components were excluded from further analyses (Table S7). We then extended this analysis to the identification of metabolites that were present in fewer than 75% of media samples generally or of cell samples, as we considered these to be of low confidence. Altogether, 14 metabolites were excluded from media and 8 from cells due to low confidence (Table S7). The remaining metabolites were included in the subsequent analyses.

### Differential accumulation patterns of metabolites over time course and EDTA treatment

We used a false discovery rate (FDR)-corrected ANOVA to determine metabolites that were significantly different in abundance between the time points. ANOVA revealed 173 and 13 metabolites that were significantly different in cells and media, respectively (Tables S8, S9). To investigate pathways associated with these differential metabolites, we mapped the set of metabolites using a custom Reference Metabolome Database with 920 compounds at BioCyc (*Streptococcus sanguinis*, Strain SK36, version 24.5: https://biocyc.org/SSAN388919/organism-summary) (Karp et al., 2019) within MetaboAnalyst by implementing overrepresentation analysis with Fisher's exact test and pathway topology analysis using relative-betweenness centrality (Jewison et al*.*, 2014). Pathway enrichment analysis of the 173 cellular metabolites identified no enriched metabolic pathways (nominal P-value < 0.05 as a cut off) that were enriched for a KEGG based pathway analysis (Fig. S2a; Table S10). Pathway enrichment analysis of the 13 media metabolites that were differential along the time course of EDTA treatment identified purine metabolism (nominal P-value < 0.05), and pyrimidine metabolism and d-glutamine and d-glutamate metabolism (both, nominal P-value < 0.1) (Fig. S2b; Table S11). When metabolite abundances were compared for the two post-EDTA time points vs T_-20_, it was revealed that 1, 12, 5, and 25 metabolites were increased in T_25_ and T_50_ in media and cells, respectively (Table S12, S13). Of these, only 2′-deoxyadenosine increased in both cells and media at T_50_ (Tables S12-13). When significantly decreased metabolites were compared, it was revealed that 1, 1, 9, and 18 metabolites were decreased in T_25_ and T_50_ in media and cells, respectively (Tables S12, S13). Only glutamine levels decreased in both media samples (Table S13). Eight of the nine metabolites that decreased at T_25_ in the cells were also significantly decreased at T_50_ (Table S12).

### Multivariate and hierarchical clustering analysis

**Fig. 2:**
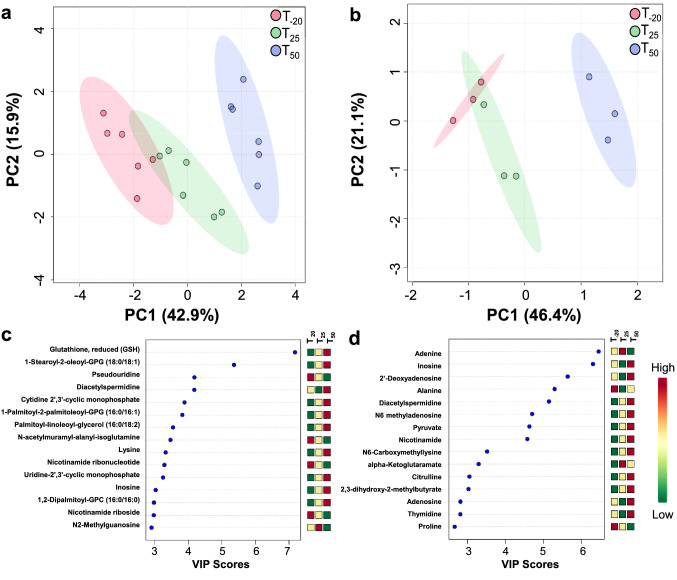
Multivariate, VIP, and time course analysis of the metabolomic changes in cells and media. Score plots of PCA displaying the separation of time-points in cells (**a**) and spent media (**b**). Cell samples n = 6; media samples n = 3. Top 15 metabolites (variables) based on VIP scores from PLS-DA analysis of cells (**c**) and spent media (**d**)

To define the metabolomic changes caused by Mn depletion, we used multivariate analysis and HCA. Using an unsupervised multivariate analysis, PCA, we observed that metabolite abundances alone were able to discriminate between the samples and explain 58.8% of the variation in the dataset by virtue of the first 2 PCs (PC1, PC2) in cells (Fig. 2a) and 67.5% in spent media (Fig. 2b).

Using supervised multivariate analysis, PLS-DA, we observed that metabolite abundances alone were able to discriminate between the samples and explain 57.1% of the variation in the dataset by virtue of the first 2 PCs (Component 1 and 2) in cells (Fig. S3a) and 57.7% in media (Figure S3b). Additionally, PLS-DA and PCA performed on all media samples explained 93.4% and 93.5% of the variation, respectively, by virtue of the first 2 PCs (Fig. S3c-d).

To identify the metabolites responsible for the discrimination among the metabolomic profiles, the variable importance in projection (VIP) score was used to select features with the most significant contribution in a PLS-DA model. VIP scores are a weighted sum of PLS weights for each variable and measure the contribution of each predictor variable to the model. Further, the VIP statistic summarizes the importance of the metabolites in differentiating the time points in multivariate space. Metabolites exhibiting high VIP scores (≥ 1.5) are the more influential variables. Our VIP analysis revealed that the top 15 metabolites for cells included lipids, cCMP, cUMP, and redox metabolites (Fig. 2c). The VIP analysis revealed that the top 15 metabolites for spent media included amino acids and organic acids (Fig. 2d). Of these VIP metabolites, seven (glutamine, adenosine, adenine, glycerate, forminoglutamate, citrulline, and orotate) were shared between cells and media across all the time points, indicating their importance.

We performed an HCA using the z-score-normalized metabolite abundances of the cellular and media metabolites, separately (Figure S4). Results indicated a clear clustering for the three time points as shown for the top 25 metabolites obtained from the ANOVA for individual sample groups. In cells, two distinct clusters were formed based on the metabolite abundances, where the upper cluster (decreased in T_50_) was represented by acetylated metabolites, purines and pyrimidines, and glutamyl dipeptides, and the bottom cluster (increased in T_50_) contained several amino acids and lipids, and cCMP, cUMP, and UTP (Fig. S4a). In media, two distinct clusters were formed based on the metabolite abundances, with the upper cluster (increased in T_50_) represented by several important metabolites such as uracil, ribose, pyruvate, nicotinamide, inosine, adenosine, guanosine, and the bottom cluster (decreased in T_50_) containing glutamine, adenine, and 3′AMP (S4b).

### Time-course analysis of cellular and media metabolites

To understand the time course-dependent changes in metabolite accumulation patterns across the three time points in this complex study design, we started with a clustering analysis. Using STEM analysis, we interrogated the time course changes of the metabolites in the cells and media. The metabolite abundances were put into 20 model clusters, which revealed differential accumulation of metabolites as a function of time. For the cells, the top two significant models were #19 (pattern 0, 1, 1, − 1; P-value 5e−115) and #18 (pattern 0, 1, −1, 0; P-value 4e−12) representing 193 and 80 metabolites, respectively (Fig. S5a; Table S14). Metabolites following the pattern in model #19 were enriched for amino acid metabolic pathways: valine, leucine and isoleucine biosynthesis and degradation, alanine, aspartate and glutamate metabolism, and glycine, serine and threonine metabolism (P-value < 0.1). Model #18 metabolites were enriched for arginine biosynthesis, arginine and proline metabolism, histidine metabolism, glyoxylate and dicarboxylate metabolism, and pyrimidine metabolism (P-value < 0.1). For the media, the top three models were #18 (0, 1, − 1, 0; P-value- 3e−59), #19 (pattern 0, 1, 1, −1; P-value- 3e−23) and #14 (pattern 1, 1, 1, 1; P-value-6e−24) representing 132, 81, and 4 metabolites, respectively (Fig. S5b and Table S15). Metabolites following the pattern in model #18 were enriched for alanine, aspartate and glutamate metabolism, amino acid metabolism, and arginine and proline metabolism. Those in model #19 were enriched for arginine biosynthesis, valine, leucine and isoleucine biosynthesis and degradation, glyoxylate and dicarboxylate metabolism, pyrimidine metabolism, alanine, aspartate and glutamate metabolism, and glycine, serine and threonine metabolism. The metabolites in model #14 included 2-deoxyadenosine, N6-methyladenosine, inosine, and nicotinamide.

### Carbohydrate metabolism and enzymatic activity in *S. sanguinis* cells in response to Mn

**Fig. 3:**
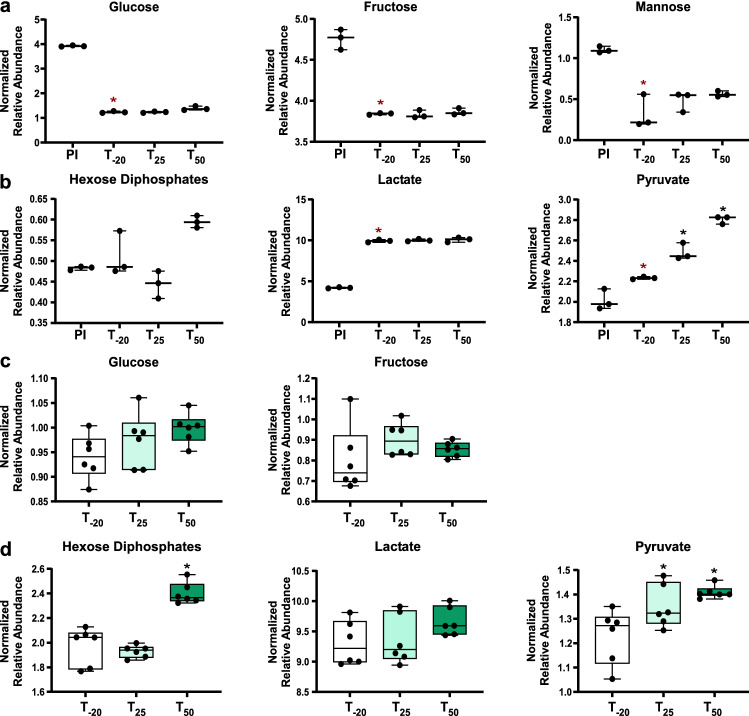
Relative abundance of carbohydrates and glycolytic intermediates in media and cells. Levels of carbohydrates in media (**a**) and cells (**c**) are depicted. Products of glycolysis in media (**b**) and cells (**d**). Whiskers indicate the range; horizontal bars represent the mean. A two-tailed t-test was used to compare the pre-inoculum (PI) media samples to post-inoculum (T_-20_). Red asterisks indicate P-value < 0.05. Spent media and cell metabolite levels were compared using one-way ANOVA with a Fisher’s least significant difference test to compare the post-EDTA (T_25_ and T_50_) samples to pre-EDTA (T_-20_). Black asterisks indicate P-value < 0.05

The levels of glycolytic byproducts in *S. sanguinis* cells and spent media were impacted by Mn depletion. Glucose, fructose, and lactate levels remained constant in cells at all three time points while pyruvate levels increased after Mn depletion (Fig. 3c, d). Lactate is known to be produced in high levels by streptococci and other lactic acid bacteria (Jakubovics et al., 2014), which explains the observed increase of lactate in the media after cellular growth. Pyruvate is produced through metabolism of sugars or amino acids. The observed increase in pyruvate levels in cells after Mn depletion (Fig. 3b) is not due to increased sugar levels, as the flow of media remained constant throughout the experiment. Most amino acid levels remained unchanged or decreased in cells after Mn depletion (Table S11). One potential explanation for the increase in pyruvate levels is that fewer pyruvate molecules were converted by pyruvate oxidase (SpxB), together with O_2_ and inorganic phosphate, into H_2_O_2_, CO_2_ and acetyl phosphate, consistent with our finding of a significant decrease in H_2_O_2_ levels after Mn depletion (Fig. 1).

Another possible explanation for the rise in pyruvate levels is the activation of pyruvate kinase by fructose-1,6-bisphosphate (FBP) (Crow & Pritchard, 1982; Jurica et al., 1998; Valentini et al., 2000). There was a significant accumulation of hexose diphosphates in cells at T_50_ and a slight increase in spent media (Fig. 3b, d). We hypothesized that the hexose diphosphate is primarily FBP and its accumulation results from the reduced activity of two potentially Mn-cofactored FBP-consuming enzymes in glycolysis/gluconeogenesis: fructose-bisphosphate aldolase (Fba) and fructose-1,6-bisphosphatase (Fbp) (Puccio et al., 2020).

**Fig. 4:**
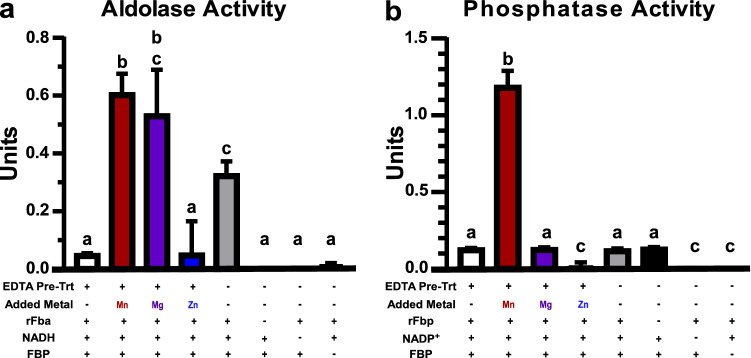
Metal dependence of FBP-metabolizing enzymes. Enzymatic activity of recombinant *S. sanguinis* enzymes were determined using coupled assays calculating the oxidation of NADH (**a**) or the reduction of NADP^+^ (**b**) spectrophotometrically at 340 nm. **a** rFba activity was measured in the presence or absence of 10 µM EDTA and 1 mM divalent metals. **b** rFbp activity was measured in the presence or absence of 10 µM EDTA with 8 mM divalent metals added. Means ± standard deviation of three replicates are depicted. Significance was determined by one-way ANOVA with a Tukey’s multiple comparisons post-test. Bars with the same letter are not significantly different from each other (P < 0.05)

To assess the metal dependence of Fba and Fbp, we generated recombinant enzymes and assayed their activity in the presence of excess metals (Fig. 4). To ensure that there were no competing metals, the recombinant enzymes (rFba and rFbp) were pre-incubated in 1 mM EDTA immediately prior to addition to the reaction mixture. The rFba enzyme showed increased aldolase activity in the presence of either 1 mM Mn or Mg, but not Zn (Fig. 4a). The negative controls had limited activity, whereas the no-EDTA control showed activity that was greater than baseline but significantly less than the Mn-treated reaction. There was a slight, but not significant, difference between the activity with Mn or Mg, indicating that in vitro, either metal increased the activity to a similar extent. The rFbp enzyme exhibited a ~ tenfold increase in phosphatase activity in the presence of 8 mM Mn but not Mg or Zn (Fig. 4b). Additionally, the presence of EDTA did not significantly affect the baseline activity and all negative controls had limited activity.

These results confirm that FBP-hydrolyzing activity is likely Mn-dependent in *S. sanguinis*. While Fba was activated by both Mn and Mg in vitro*,* Mg levels remained constant in EDTA-treated cells whereas Mn levels decreased (Fig. 1). Thus, while it is possible that Fba can utilize Mg in vivo, the evidence suggests that the increase in FBP observed post-EDTA (Fig. 3) is likely due to a Mn-dependent decrease in activity of either Fba, Fbp, or both enzymes. We hypothesize that this FBP accumulation may be responsible for the glucose-independent CcpA repression observed in the transcriptome of *S. sanguinis* after Mn depletion (Puccio et al., 2020).

Previous studies with other bacteria support a role for Mn in carbon metabolism. Mn deprivation was previously found to divert glucose from glycolysis into the pentose phosphate pathway in *S. pneumoniae* (Ogunniyi et al., 2010). *Staphylococcus aureus* was found to be more susceptible to calprotectin-mediated Mn starvation when glucose was the sole carbon source than when amino acids were also present (Radin et al., 2016). Excess Mn modulated glycolysis in *E. coli* biofilms by decreasing levels of glucose-6-phosphate and glyceraldehyde-3-phosphate (Guo & Lu, 2020). Here we provide further evidence that Mn levels impact central carbon metabolism.

### Purine and pyrimidine metabolism in Mn-deplete *S. sanguinis*

**Fig. 5:**
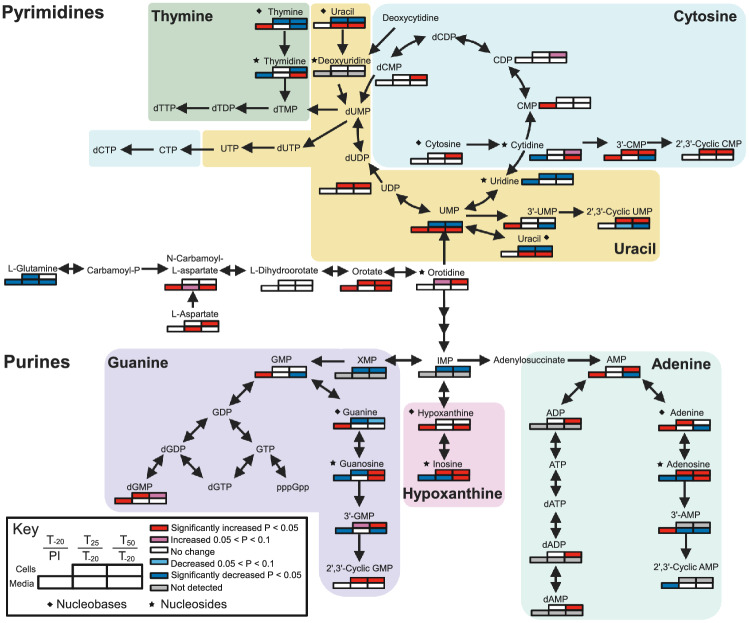
Pathways and abundance changes of nucleotide precursors resulting from Mn depletion. The direction of change in metabolite concentration is depicted in shades of red or blue, for increasing or decreasing concentration, respectively. Significance was determined by a t-test using the comparisons shown in the key. Metabolites that do not have a set of boxes were not detected in any sample. Diamonds indicate nucleobases and stars indicate nucleosides. PI, pre-inoculum. Figure created with Biorender.com

Mn is known to impact nucleotide metabolism through its role as cofactor for the aerobic ribonucleotide reductase NrdF (Makhlynets et al., 2014; Rhodes et al., 2014). Here, we observed further impacts of Mn on nucleotide metabolism. Mean levels of guanosine, inosine, and adenosine increased in both cells and media at T_50_ (Figs. 5 and S6a & e). In cells, guanine levels decreased while hypoxanthine and adenine levels were unchanged at T_50_ (Figs. 5 and S6g). This indicates that there may be blockages in the conversion of purine nucleobases into nucleosides. There are three enzymes encoded by *S. sanguinis* that can catalyze this reaction: PunA (SSA_1258), DeoD (SSA_1259), and SSA_2046. None of these enzymes have been found to use Mn (BRENDA https://www.brenda-enzymes.org/) (Jeske et al., 2019). In our recent transcriptomics study, expression of these genes was significantly decreased after Mn depletion (Puccio et al., 2020). The operon encoding *deoD* and *punA* has a carbon responsive element (*cre*) upstream (Bai et al., 2019), which is the recognition sequence for the carbon catabolite repression (CCR) regulator CcpA (Warner & Lolkema, 2003). As observed in Puccio et al. (2020), Mn depletion results in many changes in the CcpA regulon, which may explain the repression of this operon at T_50_. Thus, this may be but one example of a non-carbon catabolite pathway impacted by Mn depletion through its effect on CCR.

### Metabolomic analysis of BHI spent media reveals metabolic interactions of *S. sanguinis* with the extracellular environment

Our purpose in conducting this study was to examine the role of Mn in *S. sanguinis* metabolism, particularly in relation to IE. While the perfect medium for such a study would have been serum or plasma, this would not have been feasible, and so we instead used another complex yet commercially accessible medium—BHI. As with plasma, BHI has glucose as its most abundant sugar (0.2% w/v in BHI and ~ 0.1% w/v in plasma). Although serum and plasma have been the subject of many metabolomic studies, we are not aware of any previous metabolomic analysis of BHI. Thus, the analysis of the pre-inoculated BHI (Tables S2 or S4) may be of interest to investigators who use this medium. Likewise, the comparison of the pre-inoculated and T_-20_ media samples tells us much concerning the metabolic and transport capabilities of *S. sanguinis* under Mn-replete conditions (Table S13).

As expected, we observed a significant decrease of glucose in spent media (Fig. 3a), indicating its utilization as carbon source. Levels of fructose and mannose significantly decreased as well (Fig. 3a), indicating that they are catabolized by cells. *S. sanguinis* encodes a number of putative sugar transport systems (Das et al., 2009). Lactate and pyruvate levels increased significantly in the media after cell growth (Fig. 3b), indicating that these products of glycolysis were secreted from cells.

Also of interest, all nucleosides were significantly decreased after *S. sanguinis* growth (Figs. 5 and S6a, b). The opposite trend was observed with nucleobases, where most were significantly increased after cell growth (Figs. 5 and S6c, d). Nucleoside transport for salvage has been characterized in many bacteria, including the related species *Lactococcus lactis* (Martinussen et al., 2010) and *Streptococcus mutans* (Webb & Hosie, 2006).

## Conclusions

In this study, we showed system-wide metabolomic changes induced in *S. sanguinis* Mn-transporter mutant cells and spent media in response to EDTA treatment over time. This study captured the Mn-responsive metabolic processes, such as dysregulations in carbohydrate and nucleotide metabolism, which may contribute to the reduction in bacterial growth rate and virulence. The decrease in available Mn likely led to the reduced activity of one or both enzymes involved in FBP utilization, resulting in an accumulation of this important glycolytic intermediate and regulatory molecule. The increase in FBP likely resulted in induction of carbon catabolite repression, which may explain the blockage of nucleobases conversion into nucleosides. In addition, we provided insights into the metabolic composition of BHI and the components streptococci may utilize from this undefined medium.

## Supplementary Information

Below is the link to the electronic supplementary material.

Supplementary file1 (PDF 1149 kb)

Supplementary file2 (XLSX 651 kb)

## Data Availability

The datasets generated and analyzed during the current study are available as Supplementary Tables S1 and S2 as provided by Metabolon, Inc.
