# Supplementary material for: Time-course analysis of Streptococcus sanguinis after manganese depletion reveals changes in glycolytic and nucleic acid metabolites
Source: Metabolomics. 2021 Apr 23;17(5):44. doi: 10.1007/s11306-021-01795-2 (PMC8064989; doi:10.1007/s11306-021-01795-2)
Supplement: Supplementary file 1 — Supplementary file1 (PDF 1149 kb) [file 11306_2021_1795_MOESM1_ESM.pdf]

Time-course analysis of *Streptococcus sanguinis* after manganese depletion reveals changes in glycolytic and nucleic acid metabolites

Supplementary Material

Tanya Puccio[1], Biswapriya B. Misra[2], Todd Kitten[1]*

[1]Philips Institute for Oral Health Research, Virginia Commonwealth University School of Dentistry, Richmond 23298, VA USA.
[2]Department of Internal Medicine, Section on Molecular Medicine, Wake Forest School of Medicine, Medical Center Boulevard, Winston-Salem 27157, NC USA.

***Correspondence:**
Dr. Todd Kitten
tkitten@vcu.edu
804-628-7010

Supplementary Methods

Metal analysis

As described in Puccio et al. (2020), 40-mL cell culture samples were collected from Δ*ssaACB* cells at the same fermentor growth time points (T_{-20}, T_{25}, T_{50}). The cells were immediately centrifuged at 3,740 x *g* for 10 min at 4°C. The supernatant was decanted and the cell pellet was washed twice with cold cPBS (PBS treated with Chelex-100 resin (Bio-Rad) for 2 h, then filter sterilized and supplemented with EDTA to 1 mM). The pellet was then divided for subsequent acid digestion or protein concentration determination. Trace metal grade (TMG) nitric acid (15%) (Fisher Chemical) was added to one portion of the pellet. The pellet was digested using an Anton Paar microwave digestion system using a modified Organic B protocol: 120°C for 10 min, 180°C for 20 min, with the maximum temperature set to 180°C. The digested samples were then diluted 3-fold with Chelex-treated dH_2O. Metal concentrations were determined using an Agilent 5110 inductively coupled plasma-optical emission spectrometer (ICP-OES). Concentrations were determined by comparison with a standard curve created with a 10 µg ml^{-1} multi-element standard (CMS-5; Inorganic Ventures) diluted in 5% TMG nitric acid. Pb (Inorganic Ventures) was used as an internal standard (10 µg ml^{-1}). The other portion of the pellet was resuspended in PBS and mechanically lysed using a FastPrep-24 instrument with Lysing Matrix B tubes (MP Biomedicals) as described previously (Rhodes et al., 2014). Insoluble material was removed by centrifugation. Protein concentrations were determined using a bicinchoninic acid (BCA) Protein Assay Kit (Pierce) as recommended by the manufacturer, with bovine serum albumin as the standard. Absorbance was measured in a black, flat-bottom 96-well plate (Greiner) using a Synergy H1 plate reader (BioTek).

H_2O_2 quantitation

Culture supernatants were collected at each time point and stored at -20°C. H_2O_2 concentration was measured using a Fluorometric Hydrogen Peroxide Assay Kit (Sigma). Standards were prepared from 3% H_2O_2 provided with the kit as recommended by the manufacturer. Fluorescence was measured in a black, flat-bottom 96-well plate (Greiner) using a Synergy H1 plate reader (BioTek).

Culture enumeration

Sample culture aliquots (1 mL) from each fermentor time point were stored at 4°C. On the same day as the fermentor run, these aliquots were sonicated for 90 s using an ultrasonic homogenizer (Biologics, Inc) to disrupt chains prior to dilution in phosphate buffered saline (PBS). Diluted cultures were plated using

an Eddy Jet 2 spiral plater (Neutec Group, Inc.). The plates were incubated for 24 h at 37°C at 0% O_2, where atmospheric composition was adjusted using a programmable Anoxomat Mark II jar filling system (AIG, Inc.) and a palladium catalyst was included in the jars.

Sample preparation for metabolomics analysis

Metabolomics samples were stored at -80°C upon shipment. Samples were accessioned into the Metabolon LIMS system and were prepared using the automated MicroLab STAR® system from Hamilton Company. Several recovery standards were added prior to the first step in the extraction process for QC purposes. Samples were extracted with methanol under vigorous shaking for 2 min (Glen Mills GenoGrinder 2000) to precipitate protein and dissociate small molecules bound to protein or trapped in the precipitated protein matrix, followed by centrifugation to recover chemically diverse metabolites. The resulting extract was divided into five fractions: two for analysis by two separate reverse phase (RP)/UPLC-MS/MS methods using positive ion mode electrospray ionization (ESI), one for analysis by RP/UPLC-MS/MS using negative ion mode ESI, one for analysis by HILIC/UPLC-MS/MS using negative ion mode ESI, and one reserved for backup. Samples were placed briefly on a TurboVap® (Zymark) to remove the organic solvent. The sample extracts were stored overnight under nitrogen before preparation for analysis.

Metabolomics data generation using Ultrahigh Performance Liquid Chromatography-Tandem Mass Spectroscopy (UPLC-MS/MS)

All methods utilized a Waters ACQUITY ultra-performance liquid chromatography (UPLC) and a Thermo Scientific Q-Exactive high resolution/accurate mass spectrometer interfaced with a heated electrospray ionization (HESI-II) source and Orbitrap mass analyzer operated at 35,000 mass resolution. The sample extract was dried then reconstituted in solvents compatible to each of the four methods. Each reconstitution solvent contained a series of standards at fixed concentrations to ensure injection and chromatographic consistency. One aliquot was analyzed using acidic positive ion conditions, chromatographically optimized for more hydrophilic compounds. In this method, the extract is gradient-eluted from a C18 column (Waters UPLC BEH C18-2.1x100 mm, 1.7 µm) using water and methanol, containing 0.05% perfluoropentanoic acid (PFPA) and 0.1% formic acid (FA). A second aliquot was also analyzed using acidic positive ion conditions, but chromatographically optimized for more hydrophobic compounds. In this method, the extract is gradient eluted from the aforementioned C18 column using methanol, acetonitrile, water, 0.05% PFPA and 0.01% FA, and is operated at an overall higher organic content. A third aliquot was analyzed using basic negative ion optimized conditions using a separate

dedicated C18 column. The basic extracts were gradient-eluted from the column using methanol and water, however with 6.5 mM ammonium bicarbonate at pH 8. The fourth aliquot was analyzed via negative ionization following elution from a HILIC column (Waters UPLC BEH Amide 2.1x1$_{50}$ mm, 1.7 µm) using a gradient consisting of water and acetonitrile with 10 mM Ammonium Formate, pH 10.8. The MS analysis alternated between MS and data-dependent MS^n scans using dynamic exclusion. The scan range varies slightly between methods, but covers approximately 70-1000 m/z. Raw data files were archived and extracted as described below.

Data Extraction and Compound Identification

Raw data were extracted, peak-identified, and QC processed using Metabolon's hardware and software. These systems are built on a web-service platform utilizing Microsoft's .NET technologies, which run on high-performance application servers and fiber-channel storage arrays in clusters to provide active failover and load-balancing. Compounds were identified by comparison to library entries of purified standards or recurrent unknown entities. Metabolon maintains a library based on authenticated standards that contains the retention time/index (RI), mass to charge ratio (*m/z)*, and chromatographic data (including MS/MS spectral data) on all molecules present in the library. Furthermore, biochemical identifications are based on three criteria: retention index within a narrow RI window of the proposed identification, accurate mass match to the library +/- 10 ppm, and the MS/MS forward and reverse scores. MS/MS scores are based on a comparison of the ions present in the experimental spectrum to ions present in the library entry spectrum. While there may be similarities between these molecules based on one of these factors, the use of all three data points can be utilized to distinguish and differentiate biochemicals. More than 4500 commercially available purified standard compounds have been acquired and registered into LIMS for analysis on all platforms for determination of their analytical characteristics. Additional mass spectral entries have been created for structurally unnamed biochemicals, which have been identified by virtue of their recurrent nature (both chromatographic and mass spectral). Putative identification of each metabolite was made based on mass accuracy (m/z), Chemical Abstracts Service (CAS), Kyoto Encyclopedia of Genes and Genomes (KEGG), Human Metabolome Database (HMDB), and LIPID MAPS identifiers.

Curation

A variety of curation procedures were performed to ensure that a high quality data set was made available for statistical analysis and data interpretation. The QC and curation processes were designed to ensure accurate and consistent identification of true chemical entities, and to remove those representing system artifacts, mis-assignments, redundancy, and background noise. Metabolon data analysts used internally-

developed visualization and interpretation software to confirm the consistency of peak identification among the various samples. Library matches for each compound were checked for each sample and corrected if necessary.

Time-course analysis of cellular and media metabolomes

For our 70 min time course, we used the Short Time series Expression Miner (STEM) tool. The following parameters were used for our analysis: no normalization of data; 0 added as the starting point; number of model profiles = 20; maximum unit change in model profiles between time points = 3. To explain the model profiles, we used an expression of -1 if levels of a metabolite decreased, 0 if levels were unchanged, and 1 if levels increased. For instance, a model profile with an expression of -1, -1, 0, represents decreased, decreased, and unchanged, levels of a given set of metabolites for the 3 time points.

Metabolic pathway and enrichment analysis

Pathway enrichment analysis was performed using MetaboAnalyst 4.0 and reported pathways are BioCyc -based (Karp et al., 2019). The Chemical Translation Service (CTS: http://cts.fiehnlab.ucdavis.edu/conversion/batch) was used to convert the common chemical names into their KEGG, HMDB, Metlin, PubChem CID, and ChEBI identifiers.

Expression and purification of recombinant enzymes

The genes encoding fructose-1,6-bisphosphate aldolase (*fba*; SSA_1992) and fructose-1,6-bisphosphatase (*fbp*; SSA_1056) were codon-optimized for expression in *Escherichia coli* (Bio Basic). The optimized genes were cloned into a pET24dTEV vector with an in-frame N-terminal His-Tag. These plasmids were transformed into BL21 (λDE3) *E. coli* cells which were then grown overnight at 37°C in Luria Broth (LB; Difco) with 100 µg/mL ampicillin. This overnight culture was used to inoculate 1 L of LB, which was incubated with shaking at 30°C until the OD_{600} reached 0.7-1.0. IPTG (100 mg) was added and cells were incubated for an additional 3 h. Bacteria were harvested by centrifugation at 7000 rpm for 10 min and the pellet was resuspended in 25 mM Tris pH 8.0. Nuclease from *Serratia marcenscens* was added to 1 mg. Bacteria were lysed using a single passage through an Avestin Emulsiflex operating at 25,000 psi. The lysate was clarified by centrifugation at 20,000 rpm for 15 min.

The clarified lysate was applied to an NTA agarose column (BioRad) with a column volume of 10 mL. The column was washed with the following buffer until the A_{280} returned to baseline: 25 mM Tris pH 8.0, 10 mM imidazole, and 0.3 M NaCl. The protein was then eluted with the same buffer, except with 100

mM imidazole. Fractions were collected and analyzed by 10% or 12% SDS gels. Fractions were pooled according to the gel.

Supplementary Figures

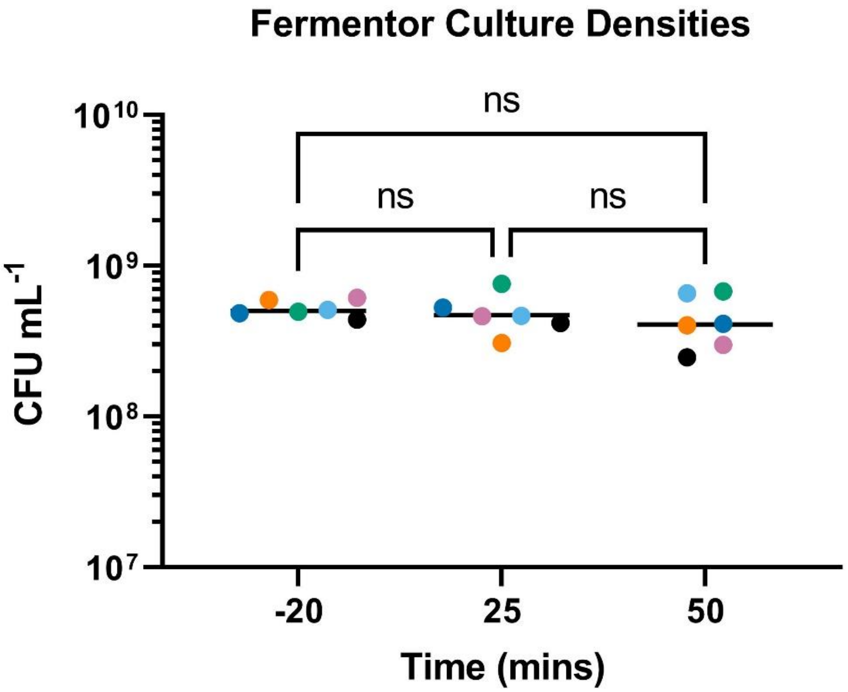

Figure S1. Densities of *S. sanguinis* Δ*ssaACB* mutant fermentor cultures subjected to metabolomic analysis

Aliquots from fermentor cultures collected at each time point were plated on BHI agar and colony forming units per mL (CFU mL^{-1}) were calculated. EDTA was added to the fermentor at T_0. The color of each circle corresponds to each of the six independent fermentor runs. Horizontal lines depict the geometric mean. Significance ($P < 0.05$) was determined by one-way ANOVA using log-transformed values.

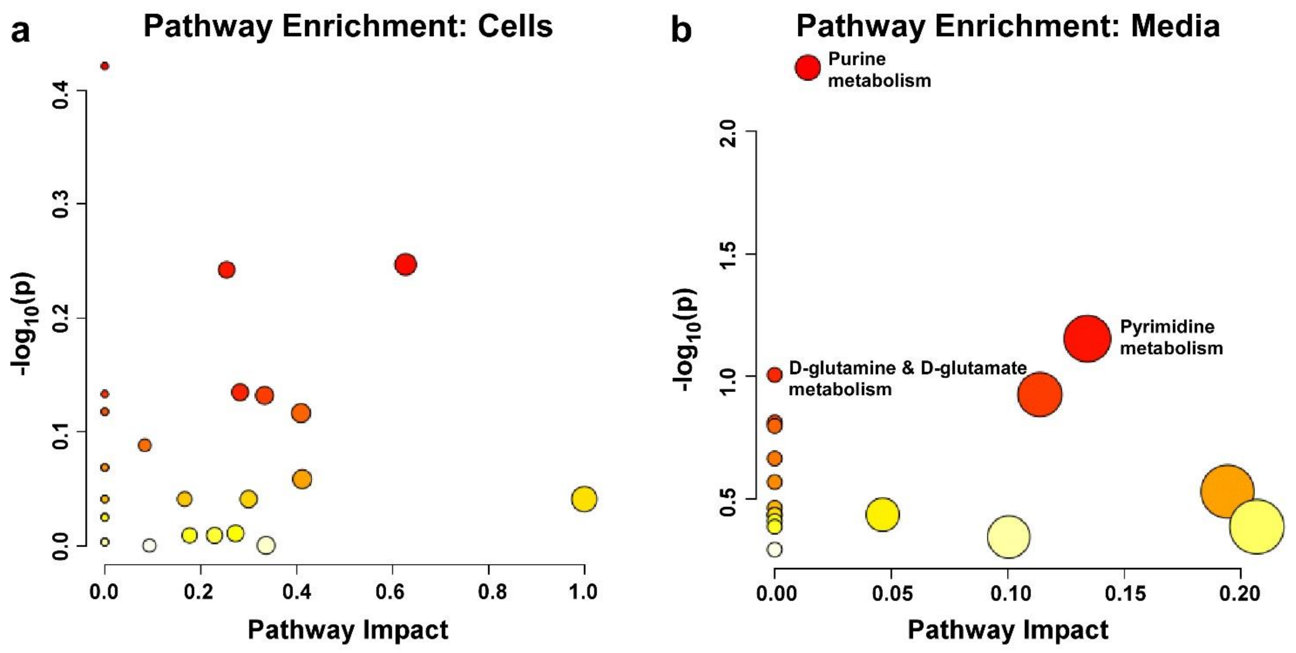

Figure S2. Pathway enrichment analysis for differential metabolites

Pathway enrichment analysis for significantly differential metabolites (ANOVA) in cells (a) and spent media (b). Red represents lower P-values (more significant in the Hypergeometric Test) and yellow/white represents greater P-values (less significant in the Hypergeometric Test). Nominal P-value < 0.05. The larger the size of the circles represents larger impact of the pathway compared to others in the pathway enrichment analysis function in MetaboAnalyst 4.0.

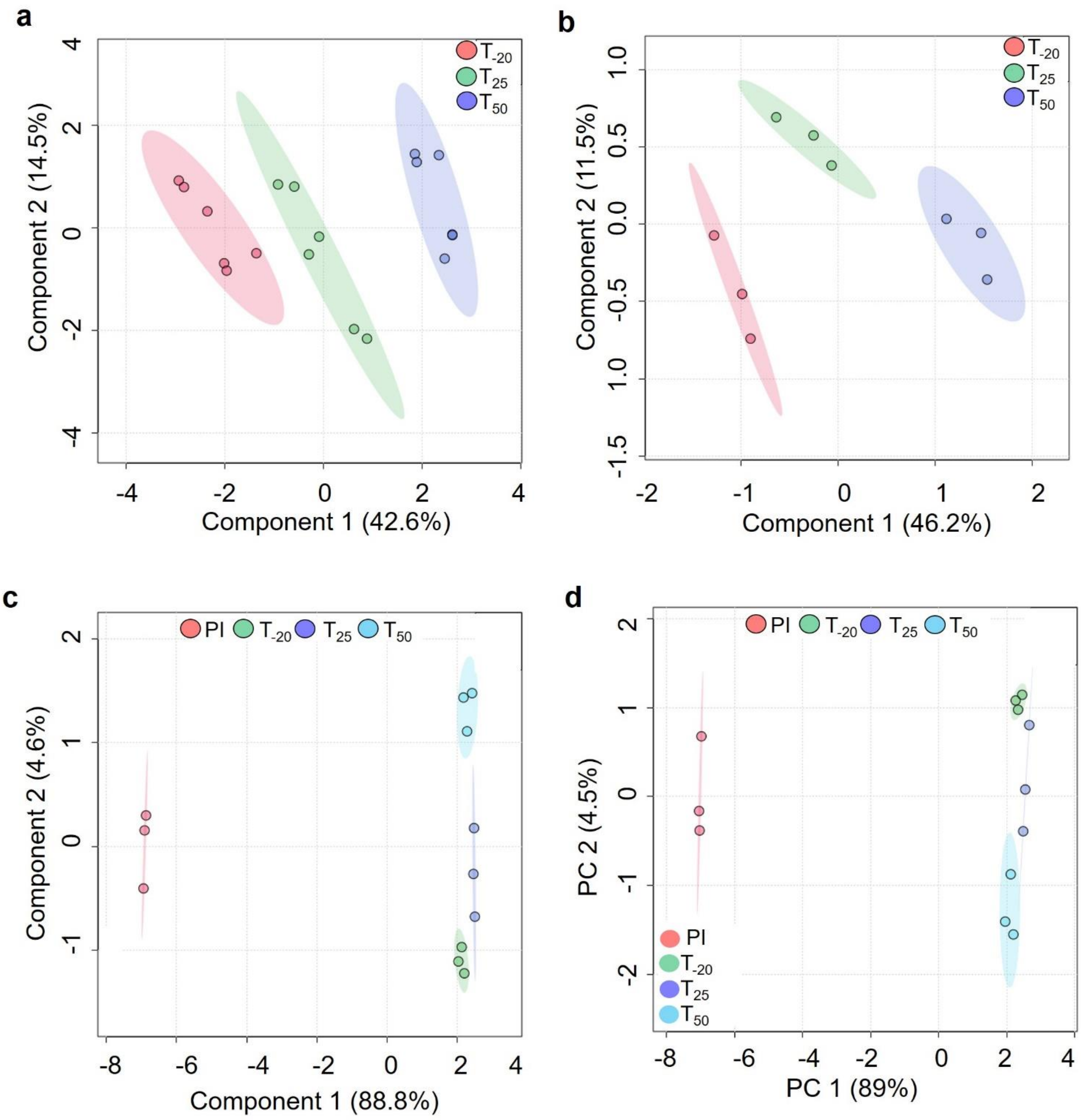

Figure S3. Multivariate (PLS-DA) analysis of the metabolomic changes in cells and media
Score plot for PLS-DA displaying the separation of time-points in cells (a) and spent media (b). Multivariate analysis of all media samples using PLS-DA (c) and PCA (d). PI; pre-inoculation.

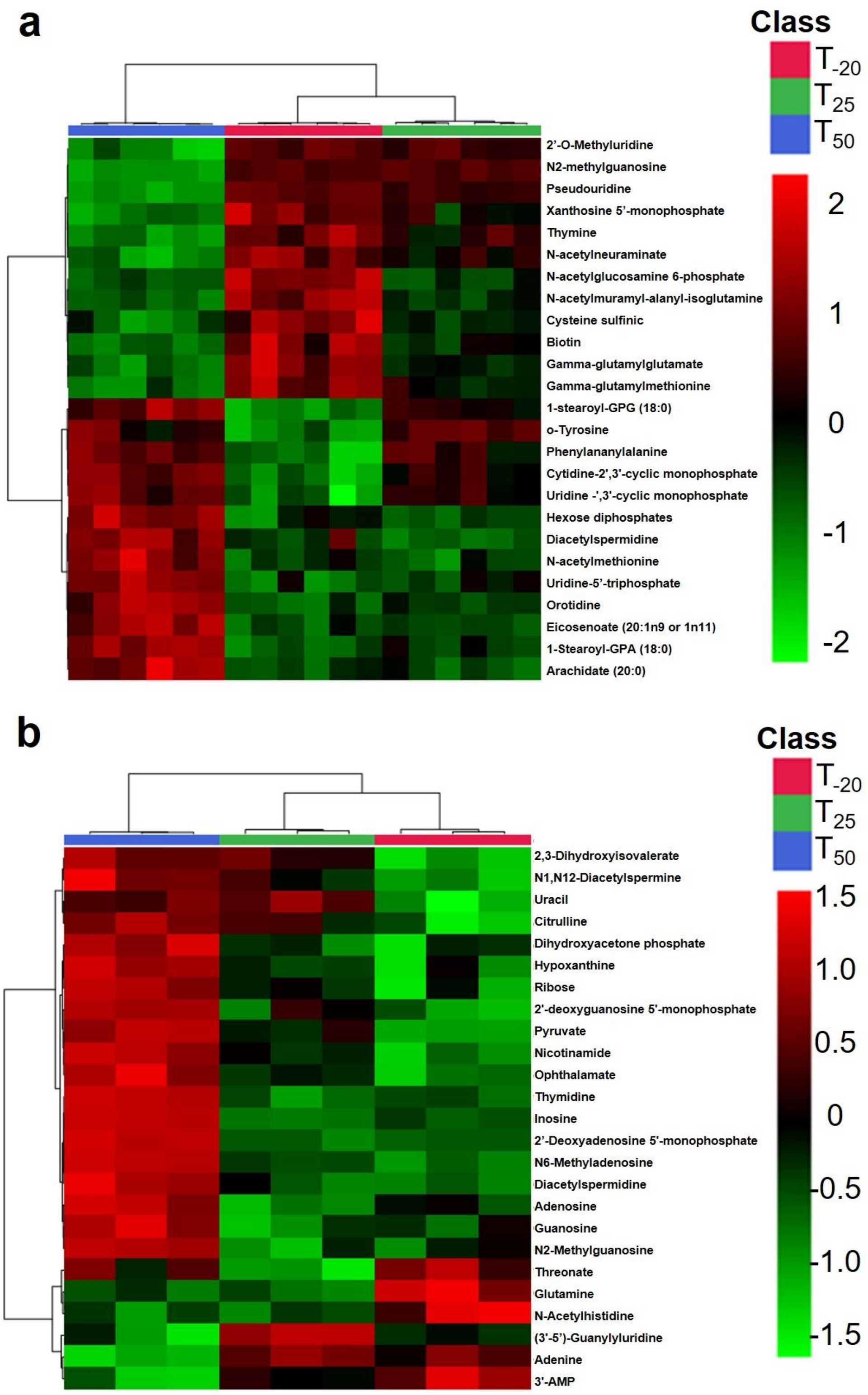

Figure S4. Hierarchical clustering analysis of the metabolites of cells and media
Top 25 ANOVA-derived differential metabolites for HCA in cells (a) and spent media (b).

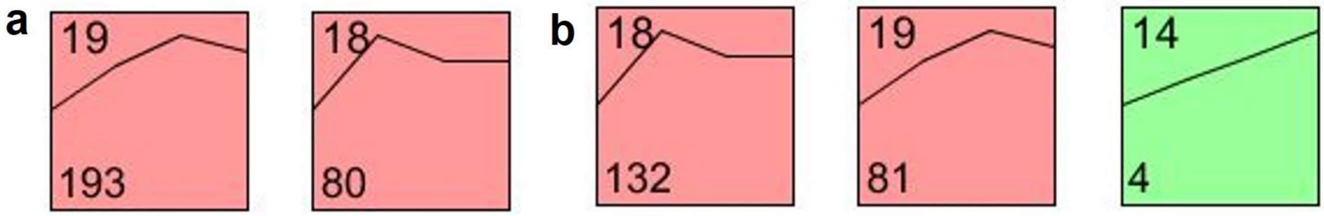

Figure S5. Time course analysis of cellular and media metabolism
Models displaying the time-dependent changes in metabolite abundance in cells (a) and spent media (b). Models #19 and #18 were statistically significant in cells among the 20 models interrogated. Models # 18, #19, and #14 were statistically significant in media.

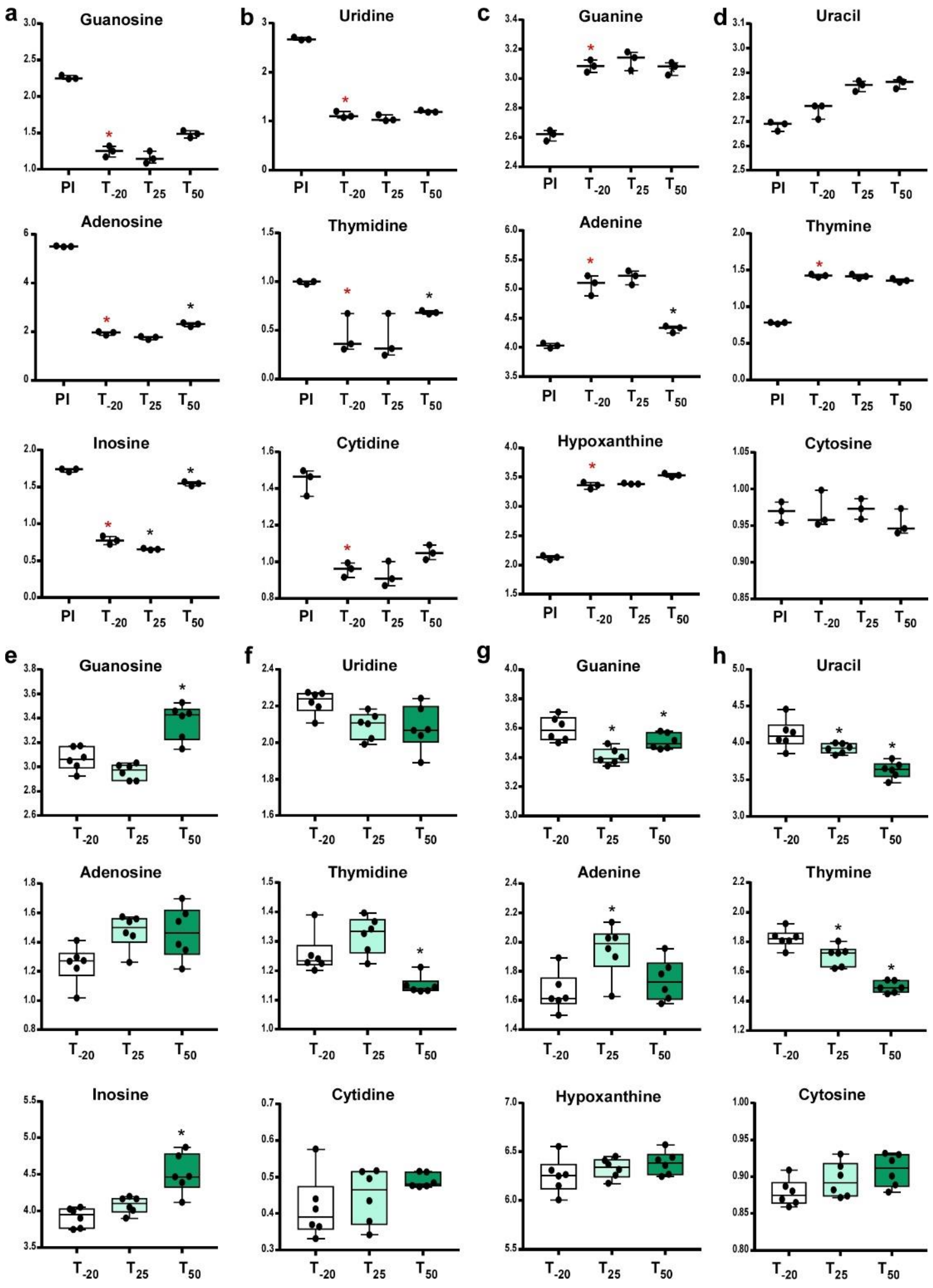

Figure S6. Changes in nucleic acid metabolites

Purine nucleosides in media (a) and cells (e). Pyrimidine nucleosides in media (b) and cells (f). Purine nucleobases in media (c) and cells (g). Pyrimidine nucleobases in media (d) and cells (h). Whiskers indicate the range; horizontal bars represent the mean. A two-tailed t-test was used to compare the pre-inoculum (PI) media samples to post-inoculum (T_{-20}). Red asterisks indicate when P-value < 0.05. Spent media and cell metabolite levels were compared using an ANOVA with a Fisher's least significant difference test to compare the post-EDTA samples to pre-EDTA. Black asterisks indicate P-value < 0.05.

Supplementary References

Karp, P.D., Billington, R., Caspi, R., Fulcher, C.A., Latendresse, M., Kothari, A., et al. (2019). The BioCyc collection of microbial genomes and metabolic pathways. *Brief Bioinform* 20(4)**,** 1085-1093. doi: 10.1093/bib/bbx085.

Puccio, T., Kunka, K.S., Zhu, B., Xu, P., and Kitten, T. (2020). Manganese depletion leads to multisystem changes in the transcriptome of the opportunistic pathogen *Streptococcus sanguinis*. *Front Microbiol* 11(2707)**,** 592615. doi: 10.3389/fmicb.2020.592615.

Rhodes, D.V., Crump, K.E., Makhlynets, O., Snyder, M., Ge, X., Xu, P., et al. (2014). Genetic characterization and role in virulence of the ribonucleotide reductases of *Streptococcus sanguinis*. *J Biol Chem* 289(9)**,** 6273-6287. doi: 10.1074/jbc.M113.533620.
